# Disrupted Regional Cerebral Blood Flow in Children With Newly-Diagnosed Type 1 Diabetes Mellitus: An Arterial Spin Labeling Perfusion Magnetic Resonance Imaging Study

**DOI:** 10.3389/fneur.2020.00572

**Published:** 2020-06-19

**Authors:** Jiawen Song, Shihan Cui, Yaomeng Chen, Xinjian Ye, Xiaoyan Huang, Haiyan Su, Yongjin Zhou, Xiaozheng Liu, Wei Chen, Xiaoou Shan, Zhihan Yan, Kun Liu

**Affiliations:** ^1^Department of Radiology, The Second Affiliated Hospital and Yuying Children's Hospital of Wenzhou Medical University, Wenzhou, China; ^2^Department of Pediatric Endocrine, The Second Affiliated Hospital and Yuying Children's Hospital of Wenzhou Medical University, Wenzhou, China; ^3^Department of Psychiatry, Sir Run Run Shaw Hospital, Collaborative Innovation Center for Brain Science, Zhejiang University School of Medicine, Hangzhou, China

**Keywords:** type 1 diabetes mellitus, children, magnetic resonance imaging, arterial spin-labeling, cerebral blood flow

## Abstract

**Object:** Diabetes is associated with cerebral vascular dysfunction and increased vascular cognitive impairment. The objective of this study was to use arterial spin labeling (ASL) perfusion-weighted magnetic resonance imaging to investigate whether cerebral perfusion was changed in newly-diagnosed children with type 1 diabetes mellitus (T1DM) and the possible relationship between aberrant cerebral blood flow (CBF) with cognitive as well as clinical variables.

**Methods:** Between January 2017 and February 2018, 34 children with newly-diagnosed T1DM and 34 age, gender, and education-matched healthy controls were included. Three dimensional pseudo-continuous ASL perfusion MRI was used to evaluate CBF. A conventional T2WI sequence was added to exclude intracranial disease. Regions with CBF differences between T1DM children and the controls were detected via voxel-wise comparisons in REST software. Associations among the result of neuropsychological test, clinical variables, and CBF values of different brains were investigated by using partial correlation analysis.

**Results:** Compared with the controls, T1DM children show decreased CBF in the left calcarine and postcentral gyrus, and right precentral gyrus. The perfusion in the postcentral gyrus was positively correlated with IQ performance. No significant correlations were found between CBF and HbA1c, blood glucose level before imaging and IQ in other brain regions in T1DM children.

**Conclusion:** There is an abnormal cerebral perfusion in children with newly diagnosed T1DM. The visual and sensorimotor areas are brain areas where perfusion is prone to change at the beginning of T1DM. Our study provided clues for cerebral pathophysiological changes in the initial stage of T1DM.

## Introduction

Type 1 diabetes mellitus (T1DM) is a chronic metabolic disorder characterized by insulin deficiency and subsequent hyperglycemia, and affects nearly 15 million children in the world (1, 2). Increased blood glucose levels, regardless of poor glycemic control or undiagnosed pathology conditions, will affect many organs and systems, including the retina, kidney, and peripheral nervous system (3). The maintenance of normal brain metabolism requires a continuous supply of glucose, thus the brain is susceptible to the disturbance of glucose metabolism. Children and adolescences are more sensitive than adults to glucose fluctuations owing to the higher cerebral energy needs to sustain the rapid myelination of neurons and normal brain development. Previous studies showed that T1DM leads to impairments in various cognitive domains in children, including intelligence quotient (IQ), attention, memory, processing speed, motor, visuospatial, and executive function (4). These impairments may begin in the early stage of disease and gradually progress with age (5, 6), which will ultimately affect the quality of life and increase the burden on families and society. Therefore, a better understanding of the effects of T1DM and its metabolic disorders on children's brain is critical for promoting evidence-based pediatric management programs and positive interventions.

The pathophysiological mechanisms underlying diabetic-related cognitive impairments are complex and multifactorial. Both hypoglycemia and hyperglycemia can contribute to cognitive impairments in T1DM. Considering that changes in consciousness occur during the severe hypoglycemic episodes in T1DM, previous studies considered hypoglycemia as one of the explanations for cognitive impairment in children with T1DM. However, a large longitudinal epidemiological study did not find this association between hypoglycemia and cognitive impairment (7). Recent studies have gradually recognized the role of hyperglycemia-induced cerebral microvascular changes such as blood-brain barrier disruption (8) and cerebral autoregulation impairment (3) in diabetes-related cognitive impairment. Among them, changes in cerebral autoregulation and endothelial function that affect local cerebral blood flow are one of the important factors affecting cognitive function (9). Proper CBF is essential for maintaining normal neural activity and metabolism, and any disruption of cerebral perfusion will lead to potential brain damage and cognitive dysfunction. Previous studies showed that diabetes cause changes in cerebral perfusion. By using [^15^O] H2O and [^18^F] positron emission tomography (PET), Larissa et al. found that patients with T1DM had decreased CBF and cerebral glucose metabolism (10).

However, previous CBF studies mainly focused on CBF of the whole brain or cerebral cortex in adult diabetic patients, which may ignore abnormal cerebral perfusion in a certain brain region or voxel. In addition, the patients had a long disease duration in these studies. Hypoglycemic events often occur during the treatment of insulin. Thus, the results may be influenced not only by the long disease duration, but also by the hypoglycemic events. Cognitive changes have been shown to occur within days of T1DM diagnosis in children and adolescents (6). It is still unclear whether there is a change in cerebral perfusion in children with T1DM at the initial stage and its relationship with cognitive or activity function. A whole brain voxel-level CBF measurement may provide a sensitive and specific imaging indicator for the diagnosis and treatment of early cognitive impairment induced by T1DM.

Arterial spin labeling is a non-invasive, sensitive, and easily repeatable perfusion-weighted magnetic resonance imaging technique that uses water protons in arterial blood as an endogenous tracer. It has unique advantages over traditional imaging methods that are usually limited by radiation or exogenous contrast agents (11, 12). Recent studies have reported that ASL reflects neurovascular coupling more directly than blood oxygen-level dependent (BOLD) fMRI and permits quantitative measurement of CBF (13). With these advantages, ASL has been widely used in the studies of cerebrovascular and mental diseases.

In the present study, we applied ASL technique to investigate whether CBF was changed in children with newly-diagnosed T1DM. The relationship between cerebral perfusion, neuropsychiatric and clinical data were also measured. We hypothesized that the children with T1DM would show alterations in cerebral perfusion patterns, and the altered perfusion would be associated with cognitive scales.

## Materials and Methods

### Subjects

This study protocol was approved by the ethics committee of our hospital. Written informed consents were obtained from parents of all participants before any research procedures. Sixty-eight participants, including 34 children with T1DM and 34 age, gender, and education-matched healthy controls, were recruited from our institution between January 2017 and February 2018. All participants were righted-handed. T1DM was diagnosed according to the American Diabetes Association criteria: history of polydipsia and/or polyuria, blood glucose level above 200 mg/dL, glycated hemoglobin A1C (HbA1C) above 6.5%, low insulin (fasting insulin level <5 μIU/ml) and C-peptide level (peak C-peptide <0.2 pmol/ml), and insulin dependency. Inclusion criteria for the T1DM group are as follows: (1) first diagnosis of T1DM in the last thirty days; (2) aged from 6 to 16 years; (3) complete clinical, imaging, and intelligence quotient (IQ) data. Inclusion criteria for the control group were children and adolescents with normal fasting glucose (<110 mg/dL), normal HbA1c (<6%), and complete clinical, imaging, and IQ data. Exclusion criteria for all participants were children with a history of neurologic or psychiatric diseases, traumatic brain injury, intracranial tumors or infection, and MRI contraindications.

### Clinical Data Collection and Neuropsychological Test

HbA1c, blood glucose level, and all relevant clinical information were obtained from participants' records. The Chinese Wechsler Intelligence Scale for Children and the Wechsler Intelligence Scale for children Fourth Edition (WISC-IV) were used to measure intellectual ability, which was evaluated by a senior medical staff who majored in intelligence assessment. Due to different subtests in the two scales, full-scale IQ was only used to assess cognitive function.

### MRI Examination

The MRI measurements were conducted on a General Electric Discovery MR 750 3.0 Tesla system using an 8-channel head coil. For T1DM children with diabetic ketoacidosis (DKA), MR scans were performed at least 3 days after the resolution of DKA. Each participant was positioned comfortably in head-first supine position in the scanner, and instructed to remain as still as possible in the scanning while maintaining eyes closed, and not to make sudden movements during the image acquisition.

The MR protocol included high-resolution T1-weighted images and three-dimensional pseudo-continuous labeling (PCASL). Anatomical T1-weighted imaging was obtained using a 3D T1-BRAVO sequence with the following parameters: repetition time (TR) = 7.2 ms, echo time (TE) = 3.4 ms, number of excitations = 1, flip angle = 12°, matrix = 256 × 256, field of view (FOV) = 240 × 240 mm^2^, sagittal slices = 188, thickness/gap = 1/0 mm, voxel size = 1.0 × 1.0 × 1.0 mm.

The resting-state perfusion imaging was performed using a pseudo-continuous ASL sequence with a 3D fast spin-echo acquisition and background suppression. The parameters were as follows: slices = 100, slice thickness = 3, flip angle = 90°, FOV = 220 × 220 mm^2^, TE = 10.5 ms, TR = 5048 ms, post labeling time (PLD) = 2025 ms, number of excitations = 3, bandwidth = 62.5. From the raw ASL data, perfusion maps were created directly from the scanner. Subjects with a head motion of more than 3.0 mm translation or a rotation of more than 3.0° in any direction were excluded.

### Data Processing

All function data were performed by using SPM8 (http://www.fil.ion.ucl.ac.uk/spm) and Data Processing Assistant for Resting-State fMRI (DPARSF, http://www.restfmri.net). At first, the perfusion images were co-registered to their anatomical T1-weighted image. The co-registered T1-weighted image was then segmented into gray matter (GM), white matter (WM) and cerebrospinal fluid (CSF) using SPM8. Furthermore, the GM images were spatially normalized to the MNI template of Montreal Neurological Institute. The quantitative CBF map of each subject was warped to the template space by using the combined warping parameters from the co-registration and normalization, and was smoothed using a Gaussian kernel with a full width at half maximum of 6 mm. Global CBF was calculated as the average of the CBF values on the entire brain mask.

### Statistics

By using REST software (http://www.restfmri.net), voxel-wise comparisons between the two groups were performed using a two-sample t test on CBF maps. Multiple comparison corrections were performed using 3dClustSim in AFNI. Uncorrected *P* < 0.005, voxel > 30, corresponding to the corrected *P* < 0.05 (parameter: single voxel *P* < 0.005, FWHM = 6 mm, voxel connection radius *r* = 5 mm, iteration = 1000) is set as statistics significance. Regression analysis was performed to test the effect of these confounding factors on the final results, with age, gender, blood glucose level at imaging, and hours passed a day during the MR scan as covariates. Finally, the abnormal brain regions were located by overlaying the statistical T maps onto the AAL and Brodmann templates.

All clinical statistical analyses were performed using SPSS 19.0 (SPSS, Inc, Chicago, IL, USA). Normality was tested by Shapiro-Wilk test. Group comparison of demographic data, clinical paraments, and neuropsychological score were performed using independent two-sample *t* tests or nonparametric Mann-Whitney U test. *P* value less than 0.05 was regarded as statistically significant.

### Correlation Between Perfusion and Clinical Data

A partial correlation analysis was performed to investigate whether the perfusion was correlated with clinical variables, with age, sex, and BMI as covariates. The statistical threshold was set at *P* < 0.05.

## Results

### Clinical Characteristic and Neuropsychological Results

Age, sex distribution, education, BMI, and hours passed a day during the MR scan had no significant differences between the diabetic group and the control group (Table 1). Recent HbA1c and blood glucose level at imaging at imaging were significantly higher in the diabetic group than the control group. Moreover, no significant difference in IQ was found between the two groups.

**Table 1:** Demographic and clinical variables of two groups.

**Variables**	**Diabetic group**	**Control group**	***P* value**
*N*	34	34	
Age (years)	9.9 ± 2.4	9.9 ± 2.1	0.96
Sex [male/female (% male)]	15/19 (0.44)	15/19 (0.44)	1.00
Education (years)	3.8 ± 2.3	3.9 ± 2.4	0.94
BMI	16.0 ± 3.2	15.2 ± 1.7	0.415
HbA_1c_ (%)	13.0 ± 2.0	5.3 ± 0.3	0.00^*^
BGL at imaging (mmol/l)	12.0 ± 4.3	4.8 ± 0.4	0.00^*^
Hours passed a day during the MR scan	18.7 ± 1.6	19.2 ± 1.8	0.24
Hypoglycemic event	0	-	-
Full-scale IQ	111 ± 17	103 ± 15	0.179

*N, number; BMI, body mass index; BGL, blood glucose level; IQ, intelligence quotient; HbA_1c_, glycosylated hemoglobin*.

*The data were presented as mean ± SD*.

* *Significant different compared with control group, P < 0.05*.

### CBF Changes

**Figure 1:**
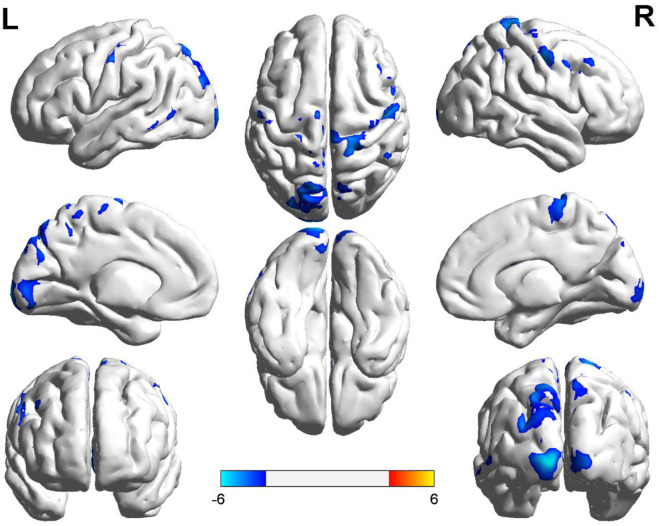
Group differences of CBF between the two groups. Compared to the controls, children with type 1 diabetes mellitus exhibited significant lower CBF in the right precentral gyrus, and the left calcarine and postcentral gyrus (cool color). Color scale denotes the T value. R, right; L, left. CBF, cerebral blood flow.

Compared with the controls, T1DM children showed decreased CBF in the left calcarine and postcentral gyrus, and right precentral gyrus (Table 2, Figure 1). However, there was no increase in CBF in any brain area in T1DM children.

**Table 2:** CBF differences between the two groups.

**Brain region**	**Brodmann area**	**Voxels number**	**MNI coordinate**			^*^T value
			***X***	***Y***	***Z***	
Calcarine_L	17	1831	−9	−105	0	−6.83
Precentral_R	4	832	51	−12	42	−5.28
Precentral_R	6	212	57	9	30	−4.72
Postcentral_L	3	132	−57	−12	48	−4.83

*MNI, Montreal Neurological Institute*.

* *T value:the extent of decline in CBF in children with type 1 diabetes compared with the children without diabetes*.

### Correlations Between CBF and Clinical Variables

**Figure 2:**
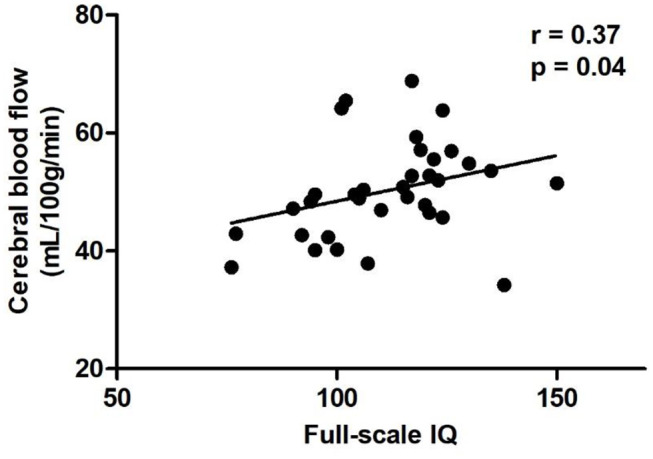
Scatter plots of the full-scale IQ compared to CBF of the left postcentral gyrus in the diabetic group. Relative CBF in the postcentral gyrus was significantly correlated with IQ scores (*r* = 0.37, *P* = 0.04). CBF, cerebral blood flow.

In the diabetic group, positive correlation was found between CBF and IQ in left postcentral gyrus (Table 3, Figure 2), while no significant correlations were found between CBF and HbA1c, blood glucose level at imaging, and hours passed a day during the MR scan in above abnormal brain regions (Table 3).

**Table 3:** Relationship between CBF and clinical data in the diabetic group.

**Brain region**	**HbA_1C_**		**BGL at imaging**		**Hours passed a day during the MR scan**		**IQ**	
	***r*^a^**	***P***	***r*^a^**	***P***	***r*^a^**	***P***	***r*^a^**	***P***
Calcarine_L	0.21	0.25	−0.16	0.53	−0.31	0.10	0.01	0.98
Precentral_R	0.20	0.29	−0.11	0.59	0.03	0.86	0.28	0.12
Precentral_R	0.27	0.14	−0.04	0.85	−0.11	0.56	0.09	0.64
Postcentral_L	0.23	0.22	−0.25	0.18	−0.24	0.20	0.37	0.04

*BGL, blood glucose level; IQ, intelligence quotient; HbA_1c_, glycosylated hemoglobin*.

a * +: increase. –: decrease*.

## Discussion

In the present study, we investigated CBF changes in newly-diagnosed T1DM children by using ASL MRI. Compared with healthy controls, children with T1DM exhibited reduced CBF in the left calcarine and postcentral gyrus, and right precentral gyrus. In addition, CBF of left postcentral gyrus was positively correlated with IQ in the diabetic group.

Previous studies showed that microvascular disease and neurodegenerative changes occurred in the visual pathway in patients with diabetes before substantial changes in the retina (14). In this study, decreased CBF of the calcarine, as the primary visual cortex, was found in T1DM children. Such hypoperfusion pattern in the visual regions was also reported in T2DM (12, 15, 16). One 3D PCASL study showed that the decreased binocular visual acuity was correlated with prolonged bolus arrival time and suggested that arterial blood supply declined may be a potential mechanism of visual impairment (16). In addition, a large number of studies have found that other ocular diseases that cause serious retinal damage, such as glaucoma, are also related to the reduction of CBF in the visual cortex (17). Therefore, we hypothesized that the hypoperfusion of the calcarine at the initial stage may be a potential mechanism of visual impairment in patients with T1DM in the future. However, the patients in this study did not have any retinopathy or clinical symptoms. Thus, follow-up of these patients is needed to determine the clinical significance of these findings.

Both the postcentral and the precentral gyrus are brain regions susceptible to diabetes. The postcentral gyrus is one part of the sensorimotor network, and is the main sensory receptive area for sense of touch, proprioception, pain, and temperature, which is also involved in developing the motor plan (18, 19). fMRI studies also found that diabetes patients showed decreased brain activity in the left postcentral gyrus (18). Selvaraiah et al. found that diabetes patients showed decreased cortical thickness of the postcentral gyrus (20). Except the brain activity and cortical thickness, this study showed that the perfusion of postcentral gyrus was also altered. The precentral gyrus is a part of premotor area and the supplementary motor area (SMA), which integrate the incoming sensory information from the outside world and the current state of body, formulating a range of complex, coordinated and detailed movement programming that sent to the primary cortex to form the final movement (19, 21, 22). Although the mechanism of movement is complex and is not fully recognized, the existed studies suggested that changes in neurovascular coupling and cerebral perfusion patterns in the motor region are associated with motor development, and even microvascular pathologies are considered to promote motor dysfunction. Tarantini et al. found that neurovascular uncoupling promoted subclinical gait abnormalities and cognitive impairment in a mouse model (23). Farzaneh et al. also found a decrease in neurovascular coupling in elderly persons with slower gait speed (24). Using PCASL technique, Stefanie et al. found that limb kinetic apraxia in people with Parkinson's disease are associated with aberrant hypoperfusion of the SMA (21). Using similar technique, a reduced perfusion in the precentral gyrus (the premotor area and SMA) was found in children with T1DM in the present study. Furthermore, the motor and sensory system involve in many high-level cognitive functions such as spatial cognition, working memory, sensory-motor transformation, perception, and decision making (22). Hence, we hypothesize that hypoperfusion in left postcentral gyrus and right precentral gyrus (the sensorimotor area, premotor area and SMA) may be potential cause of sensorimotor or cognitive impairments in T1DM.

Noteworthy, asymmetrical CBF changes were showed in the present study. We speculate that this form alteration of CBF may be attributed to handedness and hemisphere specialization for various cognitive function. Previous studies had investigated regional CBF changes in the right-handed subjects with cerebrovascular diseases in the postcentral gyrus and found that the left somatosensory cortex has a dominant role in sensorimotor integration for complex finger movements (25), whereas visuospatial is lateralized to the right hemisphere (26). Previous fMRI studies also confirmed the decreased connectivity among the right precentral gyrus and bilateral lateral occipital cortices in diabetes (27). Since all the subjects in this study were right-handed, we cannot rule out the influence of cerebral dominance in the observed CBF asymmetries. Thus, a population of left-handed participants are needed in the future study.

To date, the role of the postcentral gyrus on the general intelligence is unclear. Previous studies found positive associations between cortical thickness of the postcentral gyrus with IQ (28, 29). Moreover, recent studies showed that the postcentral gyrus is a brain region that is related to general intelligence and cognitive reserve (30). In the present study, we also found a positive correlation between CBF of left postcentral gyrus and IQ in newly-diagnosed T1DM children. However, compared to the controls, the IQ of T1DM children was not significantly changed. Hence, follow-up study is still needed to confirm the relationship between CBF of the postcentral gyrus and cognition in T1DM children. HbA1c reflects blood glucose level of the preceding 6–8 weeks and is considered as an indicator of chronic hyperglycemia. In this study, we did not find any correlation between CBF and blood glucose level or HbA1c, which was inconsistent with previous findings (31). Since the duration of the disease is shorter than 4 weeks, cerebral hypoperfusion in T1DM children may be affected by acute blood glucose fluctuations rather than chronic hyperglycemia.

There are several limitations in this study. First, the sample size is limited. Due to the wide range of variability across brains, in particular, brains in children, and other possible confounding factors, a higher number of subjects should be included in order to have more significant results across brain regions, functional data and cognitive testing. Second, the current study is cross-sectional design and cannot determine the effect of altered cerebral perfusion on cognition. Subsequent longitudinal study is needed to observe change in CBF over time and its effect on brain function and structural development. Third, twenty T1DM children had DKA, and the effect of DKA cannot be ruled out. Previous animal studies showed that hyperglycemia and ketosis independently lead to a decrease in CBF (32). However, it is unclear whether there are similar findings in humans, which needs further research to confirm. Fourth, we did not assess the level of sleepiness in these children before and after MRI scan and cannot rule out the effect of sleepiness on CBF. According to the hypothesis of synaptic homeostasis, cerebral metabolic rate, and gray matter CBF should increase when awake and decrease after sleep (33, 34). Previous study showed that individuals with a higher level of drowsy may disturb the CBF of arousal-promoting and attentional regions (35). However, a recent study did not find any associations between subjective sleepiness and CBF changes (36). The inconsistent findings in these studies could be due to a large variability in characteristics of participants, experimental designs and MRI scan parameters. Although our study did not show CBF changes in the above-mentioned drowsy-related arousal-promoting and attentional regions, it still cannot rule out the effect of sleepiness on the CBF of T1DM children, which requires further study. Finally, all T1DM children received insulin therapy, which may have an impact on the results of this study.

In conclusion, the present study used PCASL approach to examine the effect of T1DM on CBF. Our results revealed that children with newly-diagnosed T1DM exhibit decreased CBF in visual, and sensorimotor regions, suggested that there is an abnormal cerebral perfusion in these children. Our study provided clues for brain pathophysiological changes in the initial stage of T1DM.

## Data Availability Statement

The raw data supporting the conclusions of this manuscript will be made available by the authors, without undue reservation, to any qualified researcher.

## Ethics Statement

The studies involving human participants were reviewed and approved by the Ethics Committee of the Second Affiliated Hospital and Yuying Children's Hospital of Wenzhou Medical University approved this study. Written informed consent to participate in this study was provided by the participants' legal guardian/next of kin. Written informed consent was obtained from the individual(s), and minor(s)' legal guardian/next of kin, for the publication of any potentially identifiable images or data included in this article.

## Author Contributions

JS and SC contributed equally to this work and analyzed and interpreted the data and wrote and revised the manuscript. WC and XS acquired and analyzed the data and revised the manuscript. XH, XY, and HS acquired data. YC, XL, and YZ analyzed the data. KL and ZY were responsible for funding and management of the project, and revised the manuscript. All authors contributed to the article and approved the submitted version.

## Conflict of Interest

The authors declare that the research was conducted in the absence of any commercial or financial relationships that could be construed as a potential conflict of interest.
